# Characterization and Incidence of the First Member of the Genus *Mitovirus* Identified in the Phytopathogenic Species *Fusarium oxysporum*

**DOI:** 10.3390/v12030279

**Published:** 2020-03-03

**Authors:** Almudena Torres-Trenas, Encarnación Pérez-Artés

**Affiliations:** Departamento de Protección de Cultivos, Instituto de Agricultura Sostenible (IAS), Consejo Superior de Investigaciones Científicas (CSIC), 14004 Córdoba, Spain; a.torres.trenas@csic.es

**Keywords:** *Fusarium oxysporum*, hypovirulence, *Mitovirus*, mycovirus dispersion, *Narnaviridae*, viral RNA

## Abstract

A novel mycovirus named Fusarium oxysporum f. sp. dianthi mitovirus 1 (FodMV1) has been identified infecting a strain of *Fusarium oxysporum* f. sp. *dianthi* from Colombia. The genome of FodMV1 is 2313 nt long, and comprises a 172-nt 5’-UTR, a 2025-nt single ORF encoding an RdRp of 675 amino acid residues, and a 113-nt 3´-UTR. Homology BlastX searches identifies FodMV1 as a novel member of the genus *Mitovirus* in the family *Narnaviridae*. As the rest of mitoviruses, the genome of FodMV1 presents a high percentage of A+U (58.8%) and contains a number of UGA codons that encode the amino acid tryptophan rather than acting as stop codons as in the universal genetic code. Another common feature with other mitoviruses is that the 5′- and 3′-UTR regions of FodMV1 can be folded into potentially stable stem-loop structures. Result from phylogenetic analysis place FodMV1 in a different clade than the rest of mitoviruses described in other *Fusarium* spp. Incidence of FodMV1-infections in the collection of *F. oxysporum* f. sp. *dianthi* isolates analyzed is relatively high. Of particular interest is the fact that FodMV1 has been detected infecting isolates from two geographical areas as distant as Spain and Colombia.

## 1. Introduction

*Fusarium oxysporum* is a plant pathogenic fungus with worldwide distribution. This pathogenic species contains a diversity of host-plant specific forms (*formae speciales*) that cause vascular diseases in a large number of economically important crops. *F. oxysporum* f. sp. *dianthi* is the *forma specialis* of *F. oxysporum* that infects the carnation (*Dianthus caryophillus*). Carnation cultivation has a great economic importance in the cut flower sector [1,2], and its easy and rapid multiplication has made it the object of numerous commercialization of cuttings at international level. Until now, the various methodologies applied to the control of vascular wilt of the carnation have been ineffective.

Mycoviruses have been reported infecting all major taxonomic groups of fungi, including phytopathogenic and entomopathogenic fungi [3,4,5,6,7,8,9,10,11,12,13,14]. Although most of the mycoviruses described are cryptic, some species identified from different viral families show variable effects on the phenotype of their host isolate, including the induction of hypovirulence. The possibility of using these hypovirulence-inducing mycoviruses as biological control agents of the diseases caused by their fungal hosts has increased the interest for them [3,4,5,6,7,11,15,16,17]. Further, the advances in new high-throughput sequencing technologies and its low cost, are the key to the identification of new mycoviruses [18].

So far, the majority of mycoviruses described have a double-stranded RNA (dsRNA) genome, but a high number of single strand RNA (ssRNA) mycoviruses have also been discovered [6,9,19]. One of the most important families of ssRNA mycoviruses is the family *Narnaviridae* [19,20], which is constituted by two genera: *Narnavirus* and *Mitovirus*.

Mitoviruses have been identified in plants and in phytopathogenic and entomopathogenic fungi [8,13,14,21,22,23,24,25]. Mitoviruses have a genome formed by a single ssRNA molecule with a length from 2.3 to 2.9 kb, although they are normally identified in their replicative form as dsRNA. These mycoviruses possess a unique open reading frame (ORF) that encodes an RNA dependent RNA polymerase (RdRp) as the only protein [19,20]. All mitoviruses are non-encapsidated, and they are characterized by replicating within the mitochondria of the host [20,26]. Therefore, most of the genomes of viruses in the genus *Mitovirus*, like fungal and plant mitochondrial genomes, have a common property of A-U richness [27].

Although there are a few recent evidences that mycoviruses could be transmitted extracellularly, [17,28,29], apparently mitoviruses are only transferred by hyphal anastomosis between vegetative compatible strains (horizontal transmission), in the case of fungi, and during the production of spores and seeds (vertical transmission), in the case of fungi and plants. Mitoviruses use also mitochondria as a mean to transfer from the mother cell to the daughter cell during cell division [20,21,22,23].

Normally, mitoviruses are cryptic, but in some cases, effects on the mitochondria morphology, on the in vitro growth, and on the virulence of the host fungus have been described [23,24,25,30,31,32,33]. Induction of hypovirulence associated to the infection by a mitovirus has been described in phytopathogenic fungi such as *Botrytis cinerea* [23], *Cryphonectria. parasitica* [30], *Ophiostoma novo-ulmi* [25], *Sclerotinia homeocarpa* [32], and *Rhizoctonia solani* [33].

Ten members of the genus *Mitovirus* have been identified infecting different species of *Fusarium*: Fusarium circinatum mitovirus 1 (FcMV1), Fusarium circinatum mitovirus 2-1 (FcMV2-1), and Fusarium circinatum mitovirus 2-2 (FcMV2-2) from different *F. circinatum* strains collected in the north of Spain [34]; Fusarium coeruleum mitovirus 1 (FcoMV1) and Fusarium globosum mitovirus 1 (FgMV1) from *F. coeruleum* and *F. globosum* strains from Japan [35]; Fusarium poae mitovirus 1 (FpMV1), Fusarium poae mitovirus 2 (FpMV2), Fusarium poae mitovirus 3 (FpMV3), and Fusarium poae mitovirus 4 (FpMV4), detected in a single strain of *F. poae* from Japan [36]; and Fusarium boothii mitovirus 1 (FbMV1) from a strain of *F. boothii* from Ethiopia [37]. Although no data have been reported on the possible effects of these mitoviruses on the phenotype of their hosts, FgMV1 seems to reduce the mycotoxin production of its host fungus [35].

In this work, we have analyzed a collection of *Fusarium oxysporum* f. sp *dianthi* isolates obtained from soil samples and diseased carnation plants from different geographic origins (Spain and Colombia). Apparently, a relatively high number of the isolates analyzed showed a putative viral infection with a similar banding pattern of dsRNA. Sequencing and analysis of this dsRNA shows that it corresponds to a novel mitovirus, the first one described infecting a *F. oxysporum* strain. Incidence of infections with this mitovirus in the collection analyzed has also been determined.

## 2. Materials and Methods

### 2.1. Fungal Isolates and Culture Conditions

A collection of 269 *F. oxysporum* f. sp. *dianthi* (*Fod*) isolates obtained between 2008 and 2012 from plant and soil samples in Spain (Cádiz and Seville provinces) and Colombia (Gachancipá, Department of Cundinamarca), was used in this study (Table 1). All isolates were characterized to race and molecular group using multiplex-PCR, as described in Gómez-Lama Cabanás et al. [38] (Table 1). All strains were stored at −80 °C in glycerol, and propagated on potato dextrose agar (PDA) medium at 25 °C in the dark.

### 2.2. dsRNA Extractions

Mitovirus-infected isolates were identified in dsRNA extracts. The dsRNA-enriched extracts were prepared from 3 mg of the mycelia obtained after 7 days of growth in potato dextrose broth, as described in Lemus-Minor et al. [16]. The samples were pulverized in liquid nitrogen and suspended in 2 × STE buffer (50mM Tris-HCl, pH 7.0, 0.1M NaCl, 1mM EDTA), 10% SDS, and phenol. The total nucleic acids extracts obtained were then purified by cellulose column chromatography [39], and visualized by electrophoresis on 1% agarose gels stained with RedSafe Nucleic Acid Staining Solution (iNtRON Biotechnology, Seongnamsi Gyeonggi-do, Korea).

### 2.3. cDNA Synthesis, Cloning, and Sequencing

The dsRNA identified was purified and subjected to reverse transcription (RT) and PCR amplification using random hexamer priming (Invitrogen, Carlsbad, CA, USA). The amplified products were cloned into the vector pCR Blunt (Invitrogen, Carlsbad, California, USA) and sequenced. Partial nucleotide sequences were used to design specific primers for the amplification by RT-PCR of the regions not covered by the cDNA library clones (Supplementary Table S1). The terminal sequences were obtained by Single Primer Amplification Technique (SPAT), using T4 RNA ligase oligonucleotide-mediated amplification as described by Xie et al. [40]. Every region of the genome was determined by sequencing of at least 5 independent clones. Assembly of the sequences was performed using the software Geneious 8.1.5 package (Biomatters).

### 2.4. Molecular and Phylogenetic Analysis

To elucidate its genomic structure, the sequence obtained was analyzed using the software Lasergene SeqBuilder™ Version 7.0.0 (DNASTAR^®^Inc., Madison, USA). The 5′- and 3′- non coding regions (UTRs), the start codon AUG, and the different tripeptides UGA, were also identified with this program. Multiple alignment of the amino acid (aa) sequence of the RdRp to identify the conserved motifs were performed using the program MAFFT version 7 with the default parameters [41]. The secondary structures of the 5′- and 3′- UTR were predicted using the MFOLD software (version 3.5) at the MFOLD web site [42]. Phylogenetic analysis was carried out by comparison of the multiple sequence alignments using the software Geneious 8.1.5 package (Biomatters). Phylogenetic trees were constructed using the program Tree View of Geneious 8.1.5 package, and generated by the neighbor-joining method [43] with 1000 bootstrap replicates.

### 2.5. Incidence of Mitovirus FodMV1 in the Collection Analyzed

Extracts enriched in dsRNA were obtained by cellulose column chromatography of a total of 269 *F. oxysporum* f. sp. *dianthi* isolates. Presence of mitovirus FodMV1 in some of the extracts was determined by RT-PCR using a primer pair directed to the RdRp of the virus. Primers FodMV1RT, and FodMV1F/FodMV1R, were used for the RT and the PCR, respectively (Supplementary Table S1). The synthesis of cDNA was performed using 5 μL of the dsRNA extracts and the enzyme NZY M-MuLV reverse transcriptase (NZYTech, Lisbon, Portugal). The PCR amplifications were carried out with 5 μL of the synthesized cDNA and the GoTaq^®^ DNA polymerase enzyme (Promega Corporation, Madison, WI USA). The products of the RT-PCR amplifications were analyzed by electrophoresis in 1.5% agarose gels. Amplicons obtained were purified from the gel, sequenced, and analyzed for homology with the sequence of FodMV1 using the program MAFFT v7.

## 3. Results

### 3.1. Identification of Viral Infections in a Collection of F. oxysporum f. sp. dianthi Isolates

**Figure 1:**
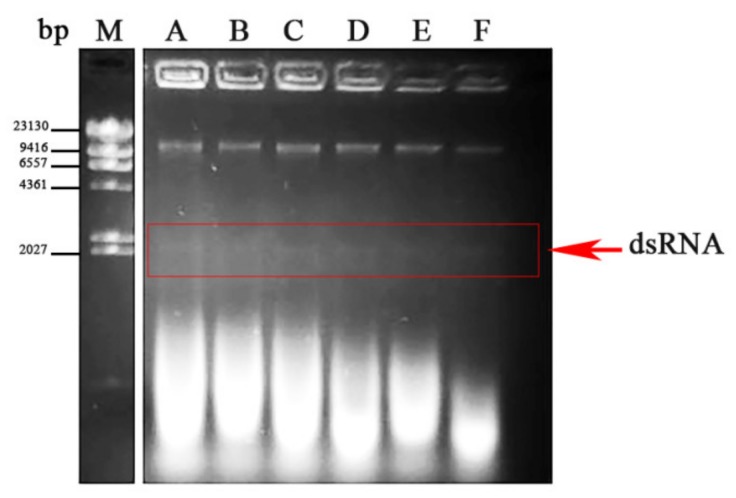
dsRNA-enriched extracts from different isolates of *Fusarium oxysporum* f. sp. *dianthi*. Agarose gel electrophoresis of the dsRNA extracts obtained by cellulose column chromatography from different isolates (A-F) of *F. oxysporum* f. sp. *dianthi.* M: molecular weight marker II (Roche Diagnostics, Rotkreuz, Switzerland).

In order to detect possible viral infections, a collection of 269 *Fusarium oxysporum* f. sp. *dianthi* (*Fod*) isolates was analyzed in this work (Table 1). All the isolates were obtained from plants and soils sampled between 2008 and 2012 in different carnation growing areas in Spain and Colombia, and had been characterized to race and molecular group by multiplex-PCR (Table 1). The presence of putative viral infections was detected in dsRNA extracts by direct observation after agarose gel electrophoresis. Forty out of the 269 isolates analyzed showed presence of a similar dsRNA element with a molecular weight of approximately 2.3 kb (Figure 1). The dsRNA nature of this element was confirmed based on its resistance to digestion with DNase I and susceptibility to degradation by RNase A at a high salt concentration (not shown). Among the infected isolates, strain *Fod* 312 was selected to characterize the putative viral dsRNA.

### 3.2. Molecular Characterization of a Novel Mitovirus Infecting F. oxysporum f.sp. dianthi

To determine the nucleotide (nt) sequence of the ~2.3 kb dsRNA element, a total of 53 clones were obtained and sequenced. A first search with some short nt sequences using the BlastX program of the NCBI, revealed that all sequences were related to mitoviral RdRp sequences. From the partial sequences obtained, specific primers were designed to fill in the gaps (Supplementary Table S1). Finally, single primer amplification technique (SPAT) was used to sequence the 5′- and 3′-terminal regions. The full-length putative genome was determined to be 2313 nt in length. A new search using BlastX program with the complete sequence showed variable homologies only with viruses in the genus *Mitovirus* (Table 2).

**Figure 2:**
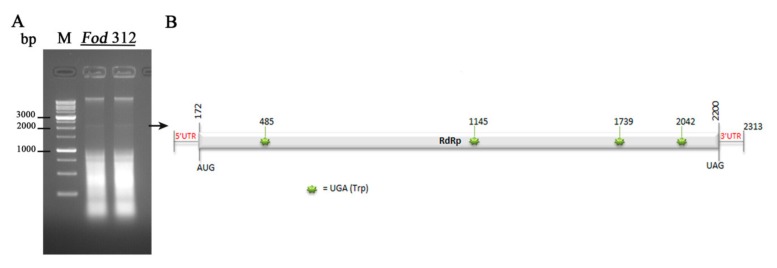
Detection of a mitovirus in isolate *Fod 312* and schematic representation of its genomic organization. (**A**) Agarose gel electrophoresis of the dsRNA-enriched extract obtained by cellulose column chromatography from isolate *Fod* 312 of *Fusarium oxysporum* f. sp. *dianthi.* Lane M: 1 kb molecular weight marker (Nippon Genetics, Japan). (**B**) Schematic representation of the genomic organization of Fusarium oxysporum f. sp. dianthi mitovirus 1 (FodMV1). The genome is 2313 bp long and contains a unique ORF that encodes an RNA dependent RNA polymerase (RdRp).

Sequence analysis revealed that the genome of the mycovirus identified had a nucleotide composition of A (29.7%), C (19.7%), G (21.3%), and U (29.1%), with an overall A+U rich content (58.8%). Sequence analysis using the DNASTAR program showed the presence of a single large ORF. This coding region started with the first AUG codon in the position 173, and finished with the UAG codon in the position 2200, and was flanked by two UTR regions of 172 nt at the 5′-UTR (nt 1-172) and 113 nt at the 3′-UTR (nt 2200–2313). This ORF encodes a putative RdRp of 675 aa residues, with a predicted molecular mass of 76.72 kDa. A schematic representation is shown in Figure 2.

**Figure 3:**
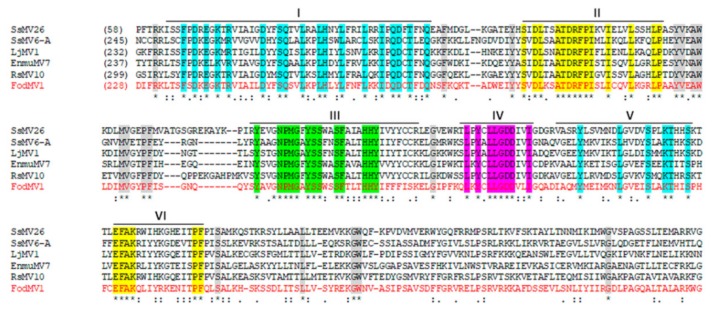
Alignments of the RNA dependent RNA polimerase (RdRp) of FodMV1 and other mitoviruses. Multiple alignment of the aa sequence of the RdRp of FodMV1 and selected viruses in the genus *Mitovirus* (see Table 3 for acronym, complete name, and GenBank accesion number of the mitoviruses used in this analysis). Alignments were performed using the program MAFFT v7 with the default parameters. Identical residues are indicated with asterisk and color shaded; colons and dots indicate conserved and semi-conserved aa residues, respectively. Conserved domains are indicated with a black line and its number (I-VI).

Alignment of the aa sequence of this complete domain with the RdRps of other mycoviruses in the genus *Mitovirus*, identified six highly conserved motifs (CM-I to VI) which are characteristic of the RdRps [25] (Figure 3). A total of six UGA codons, four in the coding sequence and two in the UTR regions, could be found throughout the complete sequence (Supplementary Figure S1).

**Figure 4:**
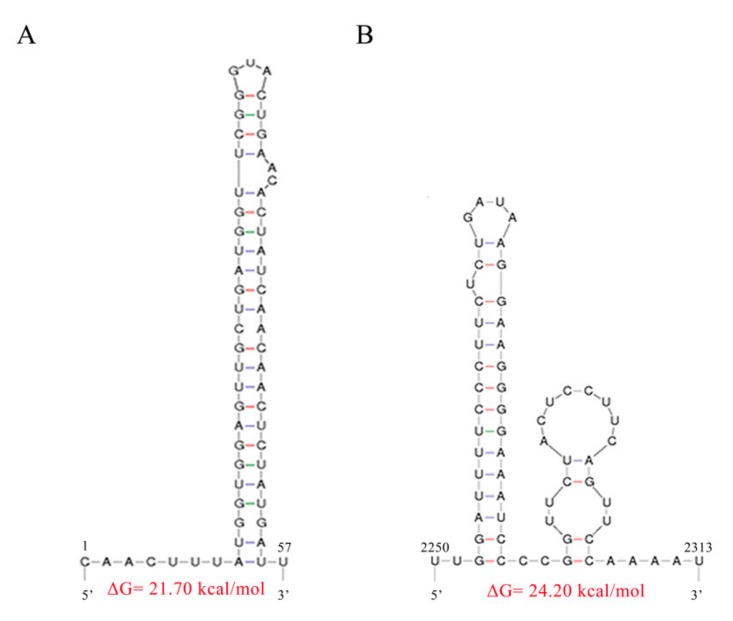
Predicted secondary structures of the terminal sequences of FodMV1. The 5´- and 3´- terminal sequences of the positive-strand of the dsRNA could be folded into potentially stable stem–loop structures. (**A**) Secondary structure of the 5′-UTR. (**B**) Secondary structure of the 3′-UTR. The MFOLD program was used to predict the secondary structure of the terminal sequences and to calculate the free energy.

Since the stem-loop structures at the 5´- and 3´- termini are characteristic of mitoviruses, we used MFOLD software to predict the potential secondary structures of the 5′- and 3′- terminal sequences of FodMV1. The result obtained revealed that the 5′- and 3′-UTRs could be folded into a predicted stable stem-loop structure (Figure 4A,B), with ΔG values of 21.70 kcal/mol for the 5´-terminal sequence, and 24.20 kcal/mol for the 3´-terminal sequence. Nevertheless, comparison of the nucleotide sequence constituting the stem-loops of FodMV1 and other mitoviruses showed no significant similarities between them.

Multiple alignment of the complete aa sequence of the RdRp of FodMV1 and the complete aa sequence of the RdRp of other mitoviruses revealed percentages of identity lower than 40% (Supplementary Table S2). Therefore, based on the rules of species demarcation criteria about mitoviruses established by the International Committee of Taxonomy for Viruses (ICTV), the mitovirus described in this work should be considered a novel member of this genus, that we have tentatively named Fusarium oxysporum f. sp. dianthi mitovirus 1 (FodMV1). The complete sequence has been deposited in the GeneBank database under the accession number MN586595.

### 3.3. Phylogenetic Relationship between FodMV1 and Other Mitoviruses.

To define the phylogenetic relationship between FodMV1 and other members in the genus *Mitovirus,* a phylogenetic analysis based on the neighbor-joining method was performed using the full length aa sequence of the viral RdRp. Due to the high number of mitoviruses identified in recent years, a number of them were selected for this analysis (Supplementary Table S2).

**Figure 5:**
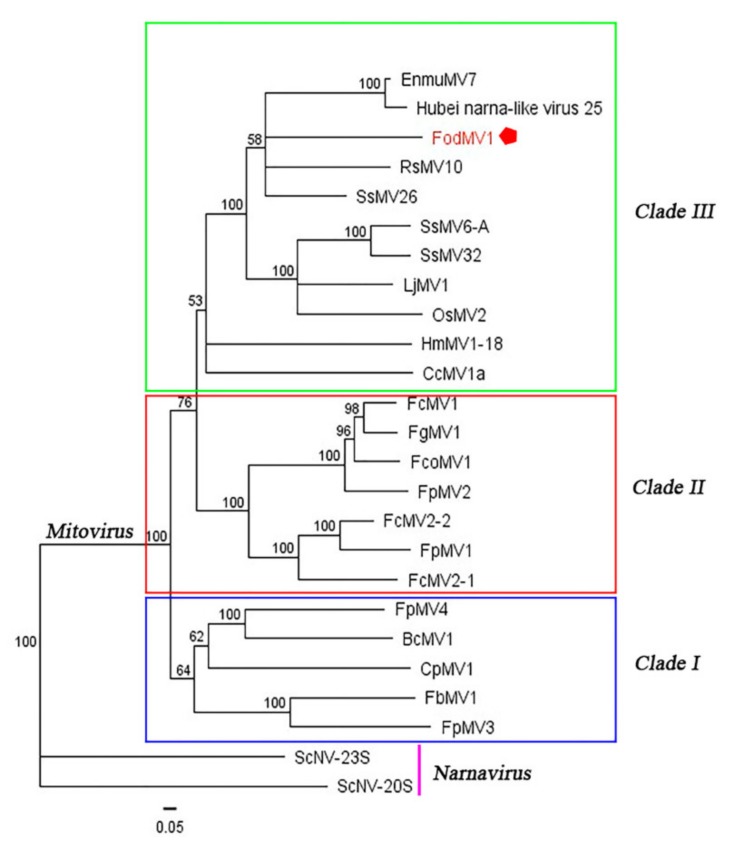
Phylogenetic analysis of FodMV1 based on the aa sequence of the RdRp. Neighbor-joining consensus tree of FodMV1 and a number of selected mitoviruses (See Supplementary Table S2) based on the full length aa sequence of the viral RdRp. Color boxes indicate the different clades obtained. The phylogenetic tree was constructed using Tree View of Geneious 8.1.5 package (Biomatters), and generated by the neighbor-joining method with 1000 bootstrap replicates.

Results obtained showed a distribution of the analyzed mitoviruses in three differentiated clades (clades I, II, and III) (Figure 5). FodMV1 placed in clade III along with other ten fungal mitoviruses, including mitoviruses of phytopathogenic fungi [Hubei narna-like virus 25, Rhizoctonia solani mitovirus 10 (RsMV10), Sclerotinia sclerotiorum mitovirus 6-A (SsMV6-A), Sclerotinia sclerotiorum mitovirus 32 (SsMV32), Sclerotinia sclerotiorum mitovirus 26 (SsMV26), Loramyces juncicola mitovirus 1 (LjMV1),Helicobasidium mompa mitovirus 1-18 (HmMV1-18), and Cryphonectria cubensis mitovirus 1a (CcMV1a)] and mitoviruses of entomopathogenic fungi [Entomorpha muscae mitovirus 7 (EnmuMV7) and Ophiocordyceps sinensis mitovirus 2 (OsMV2)] (Figure 5).

### 3.4. Incidence of Mitovirus FodMV1 in the Collection Analyzed

Of the 269 isolates analyzed by cellulose column chromatography, 40 showed presence of a similar dsRNA segment with an estimated molecular weight of ~2.3 kb. Characterization of this dsRNA element from one of the isolates (isolate *Fod* 312) showed that it was a new member of the genus *Mitovirus*, that we have named Fusarium oxysporum f. sp. dianthi mitovirus 1 (FodMV1).

**Figure 6:**
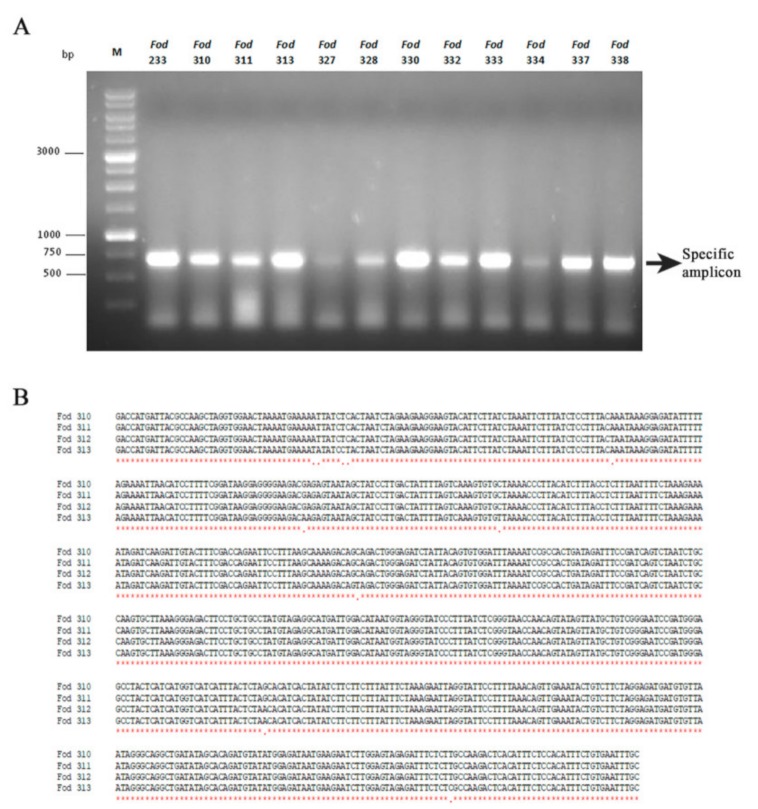
Isolates of *Fusarium oxysporum* f. sp. *dianthi* infected with mitovirus FodMV1, and alignment of the partial nucleotide sequence of the RdRp of FodMV1 from different isolates. (**A**) Specific amplicons obtained after RT-PCR of the dsRNA extracts using specific primers for the RdRp sequence of FodMV1. M: 1 kb molecular weight marker (Nippon Genetics, Japan). (**B**) Multiple alignment of the amplicons obtained by RT-PCR using specific primers for the RdRp of FodVM1 and dsRNA extracts from isolates *Fod* 310, 311, 312, and 313 of *Fusarium oxysporum* f. sp. *dianthi* from Colombia. Analysis was carried out with the program MAFFT v7.

To determine if the dsRNA element observed in the remaining 39 isolates corresponded to mitovirus FodMV1, we carried out amplifications by RT-PCR using the corresponding dsRNA extracts and specific primers designed from the nt sequence of the RdRp of FodMV1. The expected amplicon of approximately 0.7 kb was obtained as a result of the RT-PCRs in 22 of the 39 isolates analyzed (Figure 6A). These amplicons were purified from the agarose gel and sequenced. Subsequent multiple alignment between the corresponding region of the RdRp of FodMV1 and the nt sequences of the amplicons obtained from the 22 different *Fod* isolates showed nt identities between 95%–100% (Table 3). Identity was complete (100%) among the four dsRNA sequences identified in the 4 isolates from Colombia (Figure 6B), and between 95%–97% for the dsRNAs from the remaining 18 isolates, all of them originating from Spain. Regardless of the geographic origin, all FodMV1-infected isolates were of the same R2I race-group (Table 3).

## 4. Discussion

In this work, we have identified and characterized a dsRNA element found in an isolate of *Fusarium oxysporum* f. sp. *dianthi*, which represents the genome of a putative mycovirus. Homology BlastX searches revealed variable sequence similarities only with the aa sequence of other mycoviruses in the genus *Mitovirus.* Based on this result, we propose that this dsRNA (replicative form) is a novel member of the genus *Mitovirus* in the family *Narnaviridae*, that we have named Fusarium oxysporum f. sp. dianthi mitovirus 1 (FodMV1).

Due to their possible use as biological control agents [3,4,5,6,7,16,17], and to the current low cost of mass sequencing techniques [18,29], the description of new mycoviruses, in general, and of new mitoviruses in particular, is increasing considerably in recent times. Mitoviruses have been found in many phytopathogenic fungi, including both ascomycetes and basidiomycetes [23,24,25,31,32,33,34,35,36,37], and also in many entomopathogenic fungi [8,13,14]. Nevertheless, among the hundreds of mitoviruses described so far, none has been identified infecting a strain of the important phytopathogenic species *F. oxysporum*. Mitovirus FodMV1 has been detected in a strain of *F. oxysporum* f. sp. *dianthi* and, therefore, is the first mitovirus described infecting *F. oxysporum.*

Based on the molecular characterization and the genomic organization, Ghabrial and Suzuki described mitoviruses as simple naked mycoviruses whose RNA genomes encode only an RdRp protein, and that exist as RNA-RdRp nucleoprotein complexes [3]. Mitoviruses are also characterized by replicating within the mitochondria of the host [20,26,27]. Consequently, as in the case of many fungal and plant mitochondrial genomes, mitoviruses have a common property of A-U richness [27,44]. Another consequence of mitoviruses being replicated inside the mitochondria is that in the mitovirus genome, the UGA codon encodes the aa tryptophan, rather than acting as a stop codon as in the universal genetic code [26]. Our results show that the genome of FodMV1 does indeed have a high percentage of A+U (58.8%), and also contains a total of 6 UGA codons, two of them in the 5′- and 3′- non coding regions, and four in the ORF sequence. Another characteristic of mitovirus genomic RNAs is the presence of stem-loop structures at both the 5′- and 3′- ends. These structures may play important roles in mitoviral replication and in the protection of the naked ssRNA from degradation [25,44]. Moreover, stem–loop structures are common RdRp recognition sites [45,46]. In the present study, we have found that the 5′- and 3’-UTR regions of FodMV1, as in other mitoviruses, can be folded into potentially stable stem-loop structures. However, these 5’- and 3’- loop-sequences of FodMV1 showed no significant similarity with the corresponding sequences of other mitoviruses.

The genome of FodMV1 contains a single ORF that encodes an RdRp. Multiple alignment of the aa sequence of the RdRp of FodMV1 and other mitoviruses, allowed the identification of the six conserved motifs (I-VI) characteristic of the RdRps. According to Hong et al. [25], of these motifs, motif I is considered to be characteristic of the genus *Mitovirus.* Comparison of the complete nt and aa sequences of FodMV1 and other mycoviruses in the genus *Mitovirus* showed a generally limited similarity. The highest percentages of nt and aa identities were found with Sclerotinia sclerotiorum mitovirus 26 (49% and 40%, respectively), Rhizoctonia solani mitovirus 10 (44% and 38%, respectively), Entomophthora muscae mitovirus 7 ( 42% and 34%, respectively), Hubei narna-like virus (42% and 33%, respectively), Loramyces juncicola mitovirus 1 (39% and 31%, respectively), Ophiocordyceps sinensis mitovirus 2 (39% and 27%, respectively), and Sclerotinia sclerotiorum mitovirus 32 (37% and 28%, respectively). The rules of species demarcation criteria determined by the ICTV establish that the aa sequence identities of putative RdRp proteins should be less than 40% between different mitovirus species, and greater than 90% in the case of strains of the same mitovirus species [19]. According to this, FodMV1 should be considered a new species of the genus *Mitovirus*.

To provide further evidence of the relationship of FodMV1 with previously reported mitoviruses, we have carried out a phylogenetic analysis using the RdRp aa sequence. In previous phylogenetic analyses, mitoviruses were distributed into three major clades (clade I, II, and III) [24,37], and specifically, *Fusarium* mitoviruses clustered mostly in clade II [37]. Our results showed that FodMV1 clustered in a clade other than that of the rest of mitoviruses described infecting *Fusarium* spp., being more closely related to mitoviruses that infect other phytopathogenic fungi or even entomopathogenic fungi. This result seems to indicate that so far, FodMV1 is the most different among the mitoviruses described infecting *Fusarium* species.

To determine the incidence of FodMV1 infections, a total of 269 isolates of *Fusarium oxysporum* f. sp. *dianthi* obtained from different locations were analyzed. The dsRNA-enriched extracts obtained by cellulose column chromatography showed the presence of a dsRNA fragment with a molecular weight similar to that of FodMV1 in a total of 39 isolates. RT-PCR amplification of these dsRNA extracts using specific primers directed to the RdRp of FodMV1 produced the expected amplicon of approximately 0.7 kb in 22 of the 39 isolates analyzed. Sequencing of the amplicons and comparison of the sequences obtained with that of FodMV1 showed nt identities between 95% and 100% in all the cases. Identity was complete (100%) among the four dsRNA sequences identified in the 4 isolates from Colombia, and ranged between 95%–97% for the dsRNA from the rest of the isolates, all of them originating from Spain. Event thought these 0.7 kb sequences are partial, the (almost) complete identity with the correspondent sequence of mitovirus FodMV1 strongly suggests that each viral sequence corresponds to a strain of the same mitovirus FodMV1. The fact that all the virus-infected isolates were of the same R2I race-group and, therefore, of the same vegetative compatibility group [47], supports the hypothesis that a transmission of the virus could have taken place by hyphal anastomosis between isolates. Interestingly, the higher identity (100%) found among the four strains of FodMV1 from Colombia could be an indicator that the viral infection in these isolates is more recent than that present in the isolates from Spain. Presence of the same mitovirus species infecting isolates from two geographical areas as distant as Spain and Colombia could be explained by the dispersion of the viral infection through the commercialization of infected carnation cuttings between both countries.

## Figures and Tables

**Table 1:** *Fusarium oxysporum* f. sp. *dianthi* isolates analyzed for the detection of viral infections.

Isolate (s)^a^	Geographic Origin ^b^	Source	Year	Race-Group Assignation by PCR Pattern ^c^
*Fod* 108, 109, 110, 111, 120, 121	Chipiona(Ca)	Plant	2008	R1t
*Fod* 112, 113, 114, 117	Chipiona(Ca)	Plant	2008	R2I
*Fod* 118, 119	Chipiona(Ca)	Plant	2008	R2II
*Fod* 115	Chipiona(Ca)	Plant	2008	-
*Fod* 124, 127, 132, 134, 136, 138	Chipiona(Ca)	Plant	2009	R2I
*Fod* 122, 128, 130, 140, 142	Chipiona(Ca)	Plant	2009	R1t
*Fod* 183, 185, 187	Chipiona(Ca)	Plant	2010	R2I
*Fod* 181, 189, 191	Chipiona(Ca)	Plant	2010	R1t
*Fod* 179, 195,	Chipiona(Ca)	Soil	2010	R2II
*Fod* 197	Chipiona(Ca)	Soil	2010	R1t
*Fod* 144, 146, 148, 150, 158, 160, 162, 164	La Colonia(Se)	Plant	2010	R2I
*Fod* 152, 154	Lebrija(Se)	Plant	2010	R1t
*Fod* 156	Lebrija(Se)	Plant	2010	R2II
*Fod* 223, 225, 227, 229, 231, 233, 235, 237, 239, 241, 245, 247, 249, 251, 253	Chipiona(Ca)	Plant	2011	R2I
*Fod* 200, 201, 203, 205	Chipiona(Ca)	Plant	2011	R1t
*Fod* 271	Chipiona(Ca)	Soil	2011	R2I
*Fod* 256, 258, 259, 260, 261, 264, 265, 267, 269, 272, 275, 281	Chipiona(Ca)	Soil	2011	R2II
*Fod* 270, 280	Chipiona(Ca)	Soil	2011	R1t
*Fod* 207, 210, 211, 213, 215, 217, 219, 221	Lebrija(Se)	Plant	2011	R2I
*Fod* 327, 328, 329, 330, 331, 332, 333, 334, 335, 336, 341.1, 357, 358, 359, 360, 361, 364, 368, 373.2, 433, 434, 435, 436, 453	Chipiona(Ca)	Plant	2012	R2I
*Fod* 365, 452	Chipiona(Ca)	Plant	2012	R2II
*Fod* 341.2, 342, 343, 344, 345, 346, 347, 348, 349, 350, 351, 363, 366, 367, 371, 372, 373.1, 471	Chipiona(Ca)	Plant	2012	R1t
*Fod* 337, 338, 339, 340, 369, 370, 375, 437, 438, 439, 442, 443, 444, 445, 446, 451, 469	Chipiona(Ca)	Soil	2012	R2I
*Fod* 362, 376, 440, 441, 447, 448, 450, 454, 459, 463, 476	Chipiona(Ca)	Soil	2012	R2II
*Fod* 352, 353, 354, 355, 356, 374, 449, 455, 456, 457, 458, 460, 461, 462, 464, 465, 466, 467, 468, 470, 472, 473, 474, 475, 477, 478, 479, 480, 481, 482, 483, 484, 485, 486, 487	Chipiona(Ca)	Soil	2012	R1t
*Fod* 383, 384, 385, 386, 387, 388	Lebrija(Se)	Plant	2012	R2I
*Fod* 377, 378, 379, 380, 381, 382, 391, 392, 393, 394	Lebrija(Se)	Plant	2012	R1t
*Fod* 400, 402	Lebrija(Se)	Soil	2012	R2I
*Fod* 389, 390, 395, 403, 404, 405	Lebrija(Se)	Soil	2012	R2II
*Fod* 396, 397, 398, 399, 401	Lebrija(Se)	Soil	2012	R1t
*Fod* 282, 283, 291, 292, 293, 294, 295, 296, 299, 300, 301, 316, 317, 318, 320, 321, 322, 323	Colombia	Plant	2012	R1t
*Fod* 303, 304, 305, 306, 307, 308, 309, 310, 311	Colombia	Plant	2012	R2I
*Fod* 297, 298, 316, 319, 322	Colombia	Plant	2012	R2II
*Fod* 284, 287, 288, 289	Colombia	Soil	2012	R2II
*Fod* 302, 312, 313, 314, 315	Colombia	Soil	2012	R2I
*Fod* 285, 286, 290, 324, 325, 326	Colombia	Soil	2012	R1t

^a^ Isolates were analyzed for the presence of viral infections by using agarose gel electrophoresis of the dsRNA extracts obtained by cellulose column chromatography.^b^Ca = Cádiz province, Se = Sevilla province ^c^ Race-group assignation by specific-PCR amplification, as described in Gómez-Lama Cabanás et al. [38]. R1t = race 1 molecular group type, R2I = race 2 molecular group I, R2II = race 2 molecular group II. (-) Unknown data

**Table 2:** Results from BlastX homology search with Fusarium oxysporum f. sp. dianthi mitovirus 1.

Virus^a^	Acronym	Length (nt/aa Size)	Overlap (aa Identities %)	Bit score /*e*-value	Query Cover	GenBank Accesión No.
Rhizoctonia solani mitovirus 10	RsMV10	2701/752	345/563(61)	435/6e-138	70%	ALD89102.1
Entomophthora muscae mitovirus 7	EnmuMV7	2300/701	315/551(57)	385/1e-119	69%	QDH86825.1
Hubei narna-like virus 25	HNV25	2375/701	328/619(52)	357/1e-108	77%	YP_009336494.1
Loramyces juncicola mitovirus 1	LjMV1	2416/688	317/560(56)	352/7e-11	70%	AZT88622.1
Sclerotinia sclerotiorum mitovirus 6	SsMV6	2566/703	311/562(5)	325/2e-96	70%	AHF48622.1
Sclerotinia sclerotiorum mitovirus 26	SsMV26	1262/420	242/391(61)	311/1e-94	48%	AWY10984.1

^a^ The top six distinct viruses returned by BlastX are shown.

**Table 3:** Isolates of *Fusarium oxysporum* f. sp. *dianthi* infected with mitovirus FodMV1.

Host Isolate^a^	RdRp (% nt Identity)^b^	Geographic Origin^c^	Source	Year	Race group Assignation by PCR Pattern^d^
*Fod* 124	97%	Chipiona(Ca)	Plant	2009	R2I
*Fod* 233	96%	Chipiona(Ca)	Plant	2011	R2I
*Fod* 235	95%	Chipiona(Ca)	Plant	2011	R2I
*Fod* 237	95%	Chipiona(Ca)	Plant	2011	R2I
*Fod* 253	95%	Chipiona(Ca)	Plant	2011	R2I
*Fod* 219	95%	Lebrija(Se)	Plant	2011	R2I
*Fod* 215	97%	Lebrija(Se)	Plant	2011	R2I
*Fod* 221	95%	Lebrija(Se)	Plant	2011	R2I
*Fod 327*	96%	Chipiona(Ca)	Plant	2012	R2I
*Fod 328*	95%	Chipiona(Ca)	Plant	2012	R2I
*Fod 330*	96%	Chipiona(Ca)	Plant	2012	R2I
*Fod 331*	97%	Chipiona(Ca)	Plant	2012	R2I
*Fod 332*	95%	Chipiona(Ca)	Plant	2012	R2I
*Fod 333*	96%	Chipiona(Ca)	Plant	2012	R2I
*Fod 334*	97%	Chipiona(Ca)	Plant	2012	R2I
*Fod 341.1*	95%	Chipiona(Ca)	Plant	2012	R2I
*Fod 337*	97%	Chipiona(Ca)	Soil	2012	R2I
*Fod 338*	95%	Chipiona(Ca)	Soil	2012	R2I
*Fod 340*	96%	Chipiona(Ca)	Soil	2012	R2I
*Fod 310*	100%	Colombia	Plant	2012	R2I
*Fod 311*	100%	Colombia	Plant	2012	R2I
*Fod 313*	100%	Colombia	Soil	2012	R2I

^(a)^ The dsRNA from each isolate was analyzed by RT-PCR using specific primers FodMV1F/FodMV1R directed to the RdRp sequence of FodMV1. ^(b)^ Percentage of identity between the nucleotide sequences of the 0.7 kb amplicon obtained with each isolate and the correspondent sequence of FodMV1. ^(c)^ Ca = Cádiz province, Se = Sevilla province. ^(d)^ Race-group assignation by molecular markers, as described in Gómez-Lama Cabanás et al. [38]. R2I = race 2 molecular group I.

## Supplementary Materials

The following are available online at https://www.mdpi.com/1999-4915/12/3/279/s1, Figure S1: Complete nucleotide sequence of Fusarium oxysporum f. sp dianthi mitovirus 1 (FodMV1), Table S1: Specific primers and adapters used for the sequencing of FodMV1, Table S2: Amino acid identities between FodMV1 and other mitoviruses.
