# Supplementary material for: Characterization and Incidence of the First Member of the Genus Mitovirus Identified in the Phytopathogenic Species Fusarium oxysporum
Source: Viruses. 2020 Mar 3;12(3):279. doi: 10.3390/v12030279 (PMC7150889; doi:10.3390/v12030279)
Supplement: Supplementary file 1 [file viruses-12-00279-s001.zip › Figure S1.pdf]

>FodMV1

CAACUUUAUGGUGGAGUUGCUGAUGGUUCGGGUACUGAACACUAUCAACAACUCUAUGAUUAACAUUAUU
UAU**UGA**GAGGUUGAAAAACAUAAUGGCUUGCAUGAAGGUCGCAUCUUUCCUAAGGUUUUUUGUCCCCCA
AGUUCAUACCAUGUUAUUACAGCGUCAUUGUC**AUG**AAAUUAAGGAGCAUGGAUGUAUUGCGCCAAGACAG
AGGUCCCACAGCAGUGUCUGCUCGCCAAUGUCUUCUGCAUUGGUGCAAUGCCUUUCACUGUAUUUGCAAGA
UUCAAGCGUUUCUAAUCACAGAGUAUGAUCCCCGGCCCAAAUUAGAUACAAAGGAGUCAGCUUCUGGGCCA
CCACAUAAGUUGCCAGAGGAUCCCCUCCAGCUCCGACAUAGAGAAUUUGUUCCCAAAAUCCGUAAAGACCA
UGAUUACGCCAAGCUAAGGGGAGAAUUGAAUUCUGGCCCUCAUGAGGAACUCAUACAA**UGA**GACAUUGAC
UAUAAUGGUUCCUUUCUCCGUCUCUUGUACGACGAGUCCUCGGGAGGCCUGCCGAAAGCAGUGGUGGUCGC
AGAUGGUGCCUUCUGGAAAUCGAUGGGGUACCAUAGGUUACUGAGGACUCAGGAGACGGAUCAAUGGAUA
GUAGAGCAUUUGGAAGUGACUUUGAUUCACAAAUUUGGCCAUGGUGGAUGGGUAACUGGAUUGGCAGUCA
UCUGGAAACAAUCUAAGUCGGGGGCAUCCGAGGCUGUAGACCAUGAUUACGCCAAGCUAGGUGGAACUAA
AAUGAAAAAUUAUCUCACUAAUCUAGAAGAAGGAAGUACAUUCUUAUCUAAAUUCUUUAUCUCCUUUACA
AAUAAAGGAGAUAUUUUUAGAAAAUUAACAUCCUUUUCGGAUAAGGAGGGGAAGACGAGAGUAAUAGCUA
UCCUUGACUAUUUUAGUCAAAGUGUGCUAAAACCCUUACAUCUUUACCUCUUUAAUUUUCUAAAGAAAAU
AGAUCAAGAUUGUACUUUCGACCAGAAUUCCUUUAAGCAAAAGACAGCAGACUGGGAGAUCUAUUACAGU
GUGGAUUUAAAAUCCGCCACUGAUAGAUUUCCGAUCAGUCUAAUCUGCCAAGUGCUUAAAGGGAGACUUC
CUGCUGCCUAUGUAGAGGCA**UGA**UUGGACAUAAUGGUAGGGUAUCCCUUUAUCUCGGGUAACCAACAGUA
UAGUUAUGCUGUCGGGAAUCCGAUGGGAGCCUACUCAUCAUGGUCAUCAUUUACUCUAACACAUCACUAU
AUCUUCUUCUUUAUUUCUAAAGAAUUAGGUAUUCCUUUUAAACAGUUGAAAUACUGUCUUCUAGGAGAUG
AUGUGUUAAUAGGGCAGGCUGAUAUAGCACAGAUGUAUAUGGAGAUAAUGAAGAAUCUUGGAGUAGAGAU
UUCUCUUGCCAAGACUCACAUUUCUCCACAUUUCUGUGAAUUUGCUAAACAGCUUAUUUAUAGAAAAGAG
AACAUCACUCCAUUCCAAUUAUCUGCUUUGAAACAUAGUAAAUCUAGUGAUCUUAUCACUAGUCUACUAG
UUUCUUACAGAGAAAAAGGAUGGAAUGUUGCUUCUAUACCAAGUGCUGUAUCAGAUUUCUUUGGCAGAGU
CAGAGAACUUCCUUCUAGAGUUAGAAAGAAGGCAUUUGACUCAAGUGAAGUACUAAGUAACCUUAUAUAU
AUUAUAAGGGGAGACCUUCCYGCGGGCCAAGCAUUAACUGCUUUAGCUCGGAAG**UGA**GGAUACUCCUUCA
GCCUUACUGAUGAAAUUGCUACAAAUAUCAUAGCAAACAUCAUGGUUGAACUAUUUAGUGCCUCACAACC
UCAGCCUGGAAAAGGGAAACCGCUCGGAUUACUUGCCGAGAAUUUCCUUAUUUUCCUGAACGGGAUGGCU
GAGACUGGGGCCAAAGAACUAGAAGAAAACCCUGGUCTGAUGGAAUUCCACCAGAAUCCGCUGACCGAUAG
UUAUGGAUCGAUAGAGCAGCAAUACCUUGAUUUAUUAAGGAUUGCUAAGGAAAUUGACACUGUUAAACAG
GGUCAA**UGA**CCUUUGCUCUUAAGAUCUAUGACUAUCCCUCUAUCCGAUCGAUUAUUUAUCGACCGAUUUG
AGGAUACAGAGGUCAGGGCCCUGAAUUCUUUGGGAAAACCUUUGAAAGAAAGAUUUGAGGUACUAAAUCA
GUACCCUCAACUUCUUAAUUUC**UAG**GUUUUGACCCAAGGGUGCCACUCAAGGAGUGGACAAUGGGAUUAA
CCCAUUGGAUUUUCCCUUCUCUGAUAAGGAAGGGGAAAUCCCCGGUUCUACUCCUUCAGUUCCAAAAU

Supplementary Figure 1- Complete nucleotide sequence of Fusarium oxysporum f. sp dianthi mitovirus 1 (FodMV1)**.** The ``start´´ and ``stop´´ codons AUG and UAG, respectively, are indicated in green, and the six tryptophan codons UGA in red.
