# Supplementary material for: Characterization and Incidence of the First Member of the Genus Mitovirus Identified in the Phytopathogenic Species Fusarium oxysporum
Source: Viruses. 2020 Mar 3;12(3):279. doi: 10.3390/v12030279 (PMC7150889; doi:10.3390/v12030279)
Supplement: Supplementary file 1 [file viruses-12-00279-s001.zip › Table S1.pdf]

Supplementary Table 1. Specific primers and adapters used for the sequencing of FodMV1.

Name	Sequence (5′ → 3′)
[a]**FodMV1RT**	GTGGAATTCCATCAGACCAG
[a]**FodMV1F1**	GACCATGATTACGCCAAGCTA
[a]**FodMV1R2**	GCAAATTCACAGAAATGTGGAG
[a]**FodMV1F3**	TTATGGATCGATAGAGCAGC
[a]**FodMV1R4**	GAGTAGGCTCCCATCGGATT
[a]**FodMV1F5**	TCCGCCACTGATAGATTTCC
[a]**FodMV1R6**	AAGGAGTAGAACCGGGGATT
[a]**FodMV1F7**	CAGGGAGCCGCTAATAACGG
[a]**FodMV1R8**	CCATGTCAGTGTCCTCGTGA
[b]**END1**	CACGAGGACACTGACATGG
[b]**END2**	ATGGACTGAAGGAGTAGAAC
[c]**Mod1**	PO_4-GGTTCTACTCCTTCAGTCCATGTCAGTGTCCTCGTGC- NH_2

[a] Specific primers designed from the partial sequences of FodMV1.

[b] Primers for the RT (END1) and the PCR (END2) reactions, complementary to the 3′- adapter.

[c] 3′- adapter.
