# Supplementary material for: Characterization and Incidence of the First Member of the Genus Mitovirus Identified in the Phytopathogenic Species Fusarium oxysporum
Source: Viruses. 2020 Mar 3;12(3):279. doi: 10.3390/v12030279 (PMC7150889; doi:10.3390/v12030279)
Supplement: Supplementary file 1 [file viruses-12-00279-s001.zip › Table S2.pdf]

Supplementary table 2.-Amino acid identities between FodMV1 and other mitoviruses

Mitovirus	Acronym	Coding region aa identity (%)[a]	GenBank accesión no.
**Botrytis cinerea mitovirus 1**	BcMV1	15%	EF580100
**Fusarium poae mitovirus 4**	FpMV4	14%	NC030864
**Fusarium boothi mitovirus 1**	FbMV1	13%	LC425114
**Fusarium poae mitovirus 3**	FpMV3	14%	NC030863
**Entomophthora muscae mitovirus 7**	EnmuMV7	34%	QCF24458
**Hubei-Narna-like virus 25**	Hubei-Narna-like virus 25	33%	YP_009336494
**Rhizoctonia solani mitovirus 10**	RsMV10	38%	ALD89102
**Sclerotinia sclerotiorum mitovirus 26**	SsMV26	40%	AWY10984
**Sclerotinia sclerotiorum mitovirus 6-A**	SsMV6-A	29%	AWY10967
**Sclerotinia sclerotiorum mitovirus 32**	SsMV32	28%	AWY10990
**[Loramyces juncicola mitovirus 1**	LjMV1	31%	AZT88622
**Ophiocordyceps sinensis mitovirus 2**	OsMV2	27%	AZT88624
**Fusarium circinatum mitovirus 1**	FcMV1	17%	KF803546
**Fusarium globosum mitovirus 1**	FgMV1	18%	LC006128
**Fusarium coeruleum mitovirus 1**	FcoMV1	17%	LC006129
**Fusarium poae mitovirus 2**	FpMV2	17%	YP_009272899
**Fusarium circinatum mitovirus 2-2**	FcMV2-2	19%	KF803548
**Fusarium poae mitovirus 1**	FpMV1	17%	YP009272898
**Fusarium circinatum mitovirus 2-1**	FcMV2-1	16%	KF803547
**Helicobasidium mompa mitovirus 1-18**	HmV1-18	18%	AB110977
**Cryphonectria cubensis mitovirus 1a**	CcMV1a	17%	AY328476
**Cryphonectria parasitica mitovirus 1**	CpMV1	15%	L31849
**Saccharomyces 20S RNA narnavirus**	ScNV-20S	9%	AF039063
**Saccharomyces 23S RNA narnavirus**	ScNV-23s	7%	U90136

[a]Percentage of identity between the amino acid sequences of the listed mitoviruses and the amino acid sequence of FodMV1.
